# Reply to Correa et al. Comment on “Rivas et al. Unexpectedly Low Rate of Metastasis and Death Among Patients Treated for Uveal Melanoma with Brachytherapy, Vitrectomy, and Silicone Oil. *Cancers* 2025, *17*, 2683”

**DOI:** 10.3390/cancers18081224

**Published:** 2026-04-13

**Authors:** Axel Rivas, Wolfram Samlowski, Tara A. McCannel

**Affiliations:** 1 Kirk Kerkorian School of Medicine, University of Nevada, Las Vegas (UNLV), Las Vegas, NV 89106, USA; rivasa9@unlv.nevada.edu; 2 Nevada Oncology Specialists, Las Vegas, NV 89106, USA; 3 Jules Stein Eye Institute, Department of Ophthalmology, University of California, Los Angeles, Los Angeles, CA 90095, USA; mccannel@jsei.ucla.edu

We thank the leadership of the Collaborative Ocular Oncology Group (COOG) for their thoughtful comments [1] regarding our recent publication [2]. Our intention in reporting this cohort of patients was not to overstate conclusions or alter current standard clinical management. Our intention was to present real-world clinical outcomes from a consecutive cohort of patients treated at UCLA with ultrasound-directed plaque brachytherapy combined with vitrectomy and silicone oil. The survival and metastatic rates were carefully analyzed and honestly reported, and we believe they merit inclusion in the scientific record especially as they can drive further hypotheses and studies in the future. A point-by-point reply to issues raised in the letter is addressed below.

## 1. Study Design and Sample Size

We fully acknowledge the limitations of our study design, including its retrospective nature and relatively small sample size of 37 patients. These limitations were explicitly noted in the discussion section of our manuscript, and we state that our findings should be considered hypothesis-generating. A major strength of our study is that brachytherapy was delivered in a consistent fashion by a single experienced ocular oncologist. Patients were then monitored in an independent and consistent fashion by a single melanoma-specialized medical oncologist at a separate location. The median follow-up of patients reported was approximately four years, which is comparable to other studies in the field [3,4,5]. Even with these constraints, we believe it is valuable to document the outcomes of these patients.

## 2. Comparison with COOG2 Outcomes

We appreciate the COOG leadership’s reference to the COOG2 study and acknowledge its importance as the largest prospective evaluation of molecular prognostic markers in uveal melanoma [5]. However, a direct comparison between our retrospective, treatment-focused cohort and the COOG2 prognostic marker validation study is not reasonable. There are fundamental differences in study design, purpose, and patient selection. The COOG2 study was designed to validate molecular classifiers in a broad, unselected multi-institutional population compared to our analysis which focused on the outcome of a small, uniformly managed patient cohort [5]. Our observed metastasis-free survival was comparable to or exceeded expectations for patients with “high-risk” features such as monosomy 3 or gene expression profiling (GEP) Class 2. We described our results as “unexpectedly low” to contextualize outcomes related to our cohort’s risk composition. This is not to imply superiority over COOG2 or to contradict its findings. Rather we aim to highlight an interesting clinical observation that may warrant further exploration in larger, controlled studies, consistent with the spirit of hypothesis generation and scientific dialogue.

## 3. Metastatic Risk Is Driven by Molecular Alterations

We agree that the clinical staging of uveal melanoma utilizing tumor height and maximal diameter (AJCC8) represent relatively limited prognostic indicators. In fact, the majority of our patients who progressed had small- to intermediate-sized tumors. Molecular markers, including monosomy-3 and GEP, have been robustly validated by many studies including COOG and others [5,6,7]. We conclude that molecular profiling should be incorporated into future updates of AJCC uveal melanoma staging.

Our report shows that “low-risk” cytogenetic markers, such as disomy 3 or a Class 1A or B GEP score accurately predict a low probability of distant metastases (predictive value of over 90%). However, expression of “high-risk” features, such as monosomy 3 or GEP class 2 score, currently has only a modest positive predictive value in predicting which individual patients will eventually recur over many years. We believe further refinement is needed to more accurately predict patients who will actually develop distant metastases. This is supported by Harbour et al. in the COOG2 trial evaluating the prognostic value of GEP testing plus PRAME expression. [5] In this study, at 43 months median follow up, almost 60% of Class 2 patients were metastasis-free. This is comparable to the results we reported in GEP Class 2 patients.

While prognostic marker testing strongly correlated with outcome in the COOG trial, an important point is that this testing is not accurate enough to guide therapy of individual patients.

There is a significant need to develop prospective randomized trials (a) whether “low-risk” prognostic testing will allow safe de-escalation of the monitoring protocol, or (b) whether adjuvant therapy affects the potential recurrence risk of “high-risk” monosomy 3 or Class 2 patients. Development of more effective systemic treatments for uveal melanoma is sorely needed, both for metastatic disease and adjuvant treatment. Our study was not designed to prove a treatment effect on metastatic risk but to describe our institutional outcomes.

## 4. Biological Rationale

Our data raises the possibility that localized ocular treatment-related factors might further improve outcomes of uveal melanoma in ways that deserve further exploration in laboratory and clinical research. We considered differences in the management of our patients that might provide an explanation.

It is important to note that ultrasound-guided brachytherapy plaque placement may improve local tumor control. We have not identified intraocular progression in any of our patients. We recognize that the precise biological mechanism by which vitrectomy or silicone oil might influence systemic outcomes remains unproven but may merit further exploration. Prior laboratory studies demonstrated that silicone oil can attenuate radiation scatter and modify dosimetry during plaque brachytherapy [8,9]. Hypotheses we offered, such as alteration of the intraocular microenvironment or potential modification of radiation scatter, were intended to stimulate discussion, not as experimentally established explanations. As in many areas of oncology, clinical observations often precede mechanistic understanding. We believe our findings highlight the need for further translational and experimental studies to clarify whether there is any biological basis for the observed outcomes.

## 5. Clinical Benefit and Standard-of-Care

We are in full agreement that vitrectomy with silicone oil is not the current standard-of-care. At UCLA however, this combined approach with vitrectomy and silicone oil for radiation attenuation has been employed safely and effectively [10]. Visual outcomes have been published and have encouraged their continued use [11]. We are sharing our institutional experience in the hope that it will inform further research at other institutions.

## 6. Limitations

The limitations of our study, which include retrospective design and a small sample size have been noted. We appreciate that our observations may be met with appropriate skepticism, and agree that further larger, prospective, controlled studies can ultimately validate or refute our findings.

In summary, our study provides preliminary evidence that ultrasound-guided brachytherapy with vitrectomy with silicone oil during plaque brachytherapy may be associated with favorable metastasis-free survival rates for patients with uveal melanoma in a real-world setting. Our ultimate goal is to improve outcomes for patients with uveal melanoma, and we welcome continued discussion and collaboration.
